# A genome-wide scan of copy number variants in three Iranian indigenous river buffaloes

**DOI:** 10.1186/s12864-021-07604-3

**Published:** 2021-04-26

**Authors:** Maria G. Strillacci, Hossein Moradi-Shahrbabak, Pourya Davoudi, Seyed Mohammad Ghoreishifar, Mahdi Mokhber, Anoar Jamai Masroure, Alessandro Bagnato

**Affiliations:** 1 Department of Veterinary Medicine, Università degli Studi di Milano, Via dell’Università 6, 26900 Lodi, Italy; 2 Department of Animal Science, University College of Agriculture and Natural Resources, University of Tehran, Karaj, 31587-11167 Iran; 3 Department of Animal Science and Aquaculture, Dalhousie University, Truro, NS B2N5E3 Canada; 4 Department of Animal Science, Faculty of Agriculture and Natural resources, Urmia University, 11Km Sero Road, P. O. Box: 165, Urmia, 57561-51818 Iran

**Keywords:** Water Buffalo, Structural variation, Copy number variant, Biodiversity

## Abstract

**Background:**

In Iran, river buffalo is of great importance. It plays an important role in the economy of the Country, because its adaptation to harsh climate conditions and long productive lifespan permitting its farming across the Country and to convert low-quality feed into valuable milk. The genetic variability in Iranian buffalo breeds have been recently studied using SNPs genotyping data, but a whole genome Copy Number Variants (CNVs) mapping was not available. The aim of this study was to perform a genome wide CNV scan in 361 buffaloes of the three Iranian river breeds (Azeri, Khuzestani and Mazandarani) through the analysis of data obtained using the Axiom® Buffalo Genotyping Array 90 K.

**Results:**

CNVs detection resulted in a total of 9550 CNVs and 302 CNVRs identified in at least 5% of samples within breed, covering around 1.97% of the buffalo genome. and A total of 22 CNVRs were identified in all breeds and a different proportion of regions were in common among the three populations. Within the more represented CNVRs (*n* = 302) mapped a total of 409 buffalo genes, some of which resulted associated with morphological, healthy, milk, meat and reproductive traits, according to Animal Genome Cattle database.

**Conclusions:**

This work provides a step forward in the interpretation of genomic variation within and among the buffalo populations, releasing a first map of CNVs and providing insights about their recent selection and adaptation to environment. The presence of the set of genes and QTL traits harbored in the CNVRs could be possibly linked with the buffalo’s natural adaptive history together to a recent selection for milk used as primary food source from this species.

**Supplementary Information:**

The online version contains supplementary material available at 10.1186/s12864-021-07604-3.

## Background

The Asian water buffalo (*Bubalus bubalis*) and the African wild buffalo or Cape buffalo (*Syncerus caffer*) are the two main species of buffalo in the world [1]. The domestication of the Asian water buffalo with two subspecies i.e. the river (*Bubalus bubalis bubalis* 2n = 50) and the swamp (*Bubalus bubalis carabanensis* 2n = 48) buffalo, occurred about 3000–6000 years before present in the Indo–Pakistani area and in the vicinity of borders of China, respectively [2, 3]. River buffalo is common in India, Egypt, Southwest Asia and Europe, and swamp buffalo is common in China and Southeast Asia [3, 4].

According to the FAOSTAT data (http://www.fao.org/faostat/en/?#data/QA accessed 2020/10/08) the proportion of buffalo population in Iran respect to cattle species was 2.5%. A very similar data is reported by Beldman et al. [5] who analyzed the dairy farming sector in Iran indicating a proportion of 3% of buffalo heads over the total population of cattle.

In the developing countries including Iran, river buffalo breeding is nowadays recognized of great importance because of (i) the ability of buffalo to convert low-quality feed to valuable milk, (ii) adaptation to harsh climate conditions and resistance to local parasites, (iii) long productive lifespan [6], and (iv) their potential in milk and meat production performances [7]. As described by Safari et al. [7] the main breeding activities in Iranian Buffalos are carried by the Animal Breeding Center of Iran and envisage milk recording and genetic evaluation of reproducers. In Iran, there are three main buffalo breeds including Azeri (from the north-west and north), Khuzestani from west and south-west, and Mazandarani (from north) [4, 6]. The recently released buffalo SNP genotyping array has been reported as a suitable tool for studying genetic diversity of river buffalo breeds as well as a potential starting point for genome-wide association and genomic selection programs [1, 8]. The genetic variability in these three breeds have been recently studied using SNPs markers. Davoudi et al. [9] investigated the haplotypic structure and genetic diversity in Khuzestani river buffalo, while Mokhber et al. [4] evaluated the genetic structure of the Azeri and Khuzestani breeds to identify genomic regions associated to different environmental conditions and production goals. Additionally, Ghoreishifar et al. [6] identified ROH in the Azeri and Khuzestani breeds.

Copy number variants (CNVs) are a source of structural variability that have been utilized to identify genetic variability among breeds in several species. Redon et al. [10] defined a CNV as a DNA segment of one kilobase (kb) or larger that is present at a variable copy number in comparison with a reference genome. Several studies have been performed in different species, finding that CNVs are also related to phenotypic variability [11–16] as well as disease susceptibility [17, 18] describing up to 30% of the genetic variation in gene expression. Studies regarding the CNVs detection based on SNP chip data in buffalos were not available, consequently, the aim of this research was to perform a genome wide CNV mapping in samples of the three Iranian river buffalo breeds through the analysis of data obtained using the Axiom® Buffalo Genotyping Array 90 K.

## Results

The PCA and the F_ST_ results based on the SNPs genotypes confirms that the three populations are clearly differentiated (Fig. S1).

A total of 9550 CNVs (5154–53.97% – deletions and 4396–46.03% – gains) on all 24 autosomes were detected in the 361 samples (Supplementary Table S1). Table 1 reports the descriptive statistics for CNVs for each of the three buffalo populations, as well as the loss/gain ratio calculated considering the number of loss on number of gains. The largest loss/gain ratio was found in the MAZ breed (1.32) while the KHU showed a value close to the unit.

**Table 1:** Summary of statistics for CNV detected in the three Buffalo populations

Breed	N samples	N. CNV	Loss	Gain	Loss / Gain ratio	Min - Max CNV per samples (mean ± SD)	Min Length	Max Length	Mean Length
**AZE**	242	6415	3528	2887	1.22	9–39 (26.51 ± 5.66)	7492	3,484,078	110,412.3
**KHU**	100	2742	1399	1343	1.04	15–44 (27.42 ± 6)	7492	2,757,145	103,653.3
**MAZ**	19	393	227	166	1.37	11–26 (20.68 ± 4.20)	5702	1,731,686	129,681.7

**Fig. 1:**
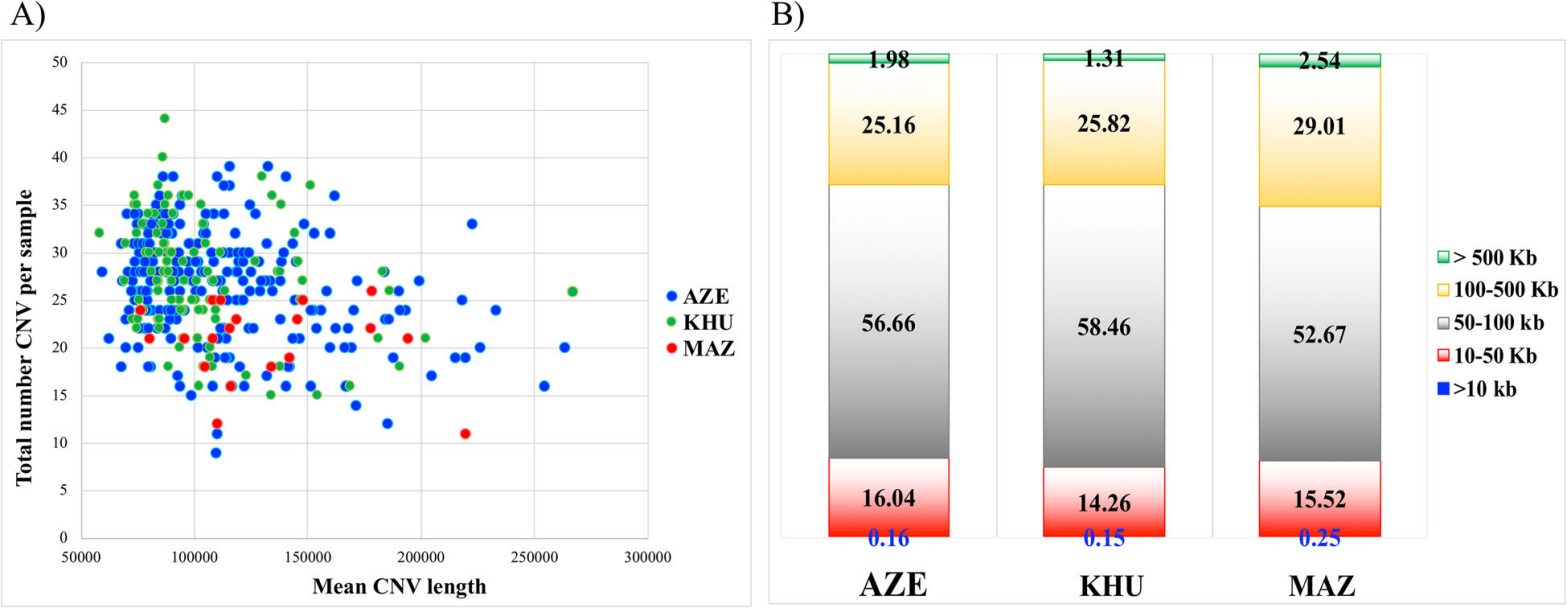
Graphical representation of CNV statistics. **a** Relationship between number and mean total length (bp) of CNV identified in each sample: Blu dots – AZE breed, Green dots KHU breed, Red dots – MAZ breed; **b** Proportion of the 5 classes of CNV Length (Green > 500 Kb, Yellow 100–500 Kb, Grey 50–100 Kb, Orange 10–50 Kb, blue <10Kb) for each of the three breeds

The graphical representations of CNV statistics are shown for each breed in Fig. 1. In details, the relationship between CNV count and the averaged total length of CNV for each individual is shown in Fig. 1a The graphical distribution allowed to identify a similar pattern of distribution for the samples belonging to the three buffalo breeds with few individuals counting a low number of CNVs with a high average length. Even when CNVs are classified according to classes of length (5 classes as in Fig. 1b legend), the three populations showed similar structure in CNVs. An exception was observed for the proportion of CNVs for the longer class of length (> 500 Kb), resulted higher in MAZ respect those identified for AZE and KHU breeds.

Overlapping CNVs across samples within population were summarized into 1678, 1060, and 257 copy number variable regions (CNVRs) for AZE, KHU and MAZ, respectively (Table 2). The Supplementary Table S2 reports the list of CNVRs found in each population, together with the samples CNV count and states.

**Table 2:** Summary of all CNVRs identified in the three Buffalo populations. Min, Max and Mean length are expressed in base pair (bp)

Breed	N. CNVRs	Loss	Gain	Complex	Singleton (^a^)	Min-Max (mean) length	Coverage (Mb) (%)^b^
AZE	1678	795	703	180	834 (49.7%)	7491-3,484,077 (137,485)	206.18 (8.32)
KHU	1060	492	485	83	583 (55%)	8300-3,431,078 (127,376)	109.60 (4.42)
MAZ	257	148	106	3	188 (73.15%)	5701-1,731,685 (123,129)	26.59 (1.07)

^a^percentage calculated on total CNVRs number; ^b^percentage calculated on total Buffalo autosome length (2478.74 Mb – https://www.ncbi.nlm.nih.gov/genome/?term=bubalus)

The total number of regions identified, as reported in Table 1, is possibly linked to the sample’s population size, especially for singleton_CNVRs that in the MAZ breed result to be a very large proportion (73%) of identified CNVRs: it is more likely, in fact, to identify singleton_CNVRs in small populations as they are those not in common among individuals.

A total of 101, 133 and 68 CNVRs (n. 302 in total, 203 non redundant regions) are the regions defined by CNVRs mapped in at least 5% of samples (n. 12 – AZE, n. 5 – KHU, and n. 2 – MAZ). Non redundant regions include pop_CNVRs (i.e. non singleton regions within population) plus the common_CNVRs (i.e. common CNVRs across populations), counting these latter only once when found in more than one population. All the following statistics and graphical representation have been obtained using the pop_CNVRs, covering 28.40 Mb (1.14%), 29.91 Mb (1.21%), and 11,44 Mb (0.46%) of the buffalo autosomes total length, respectively.

**Fig. 2:**
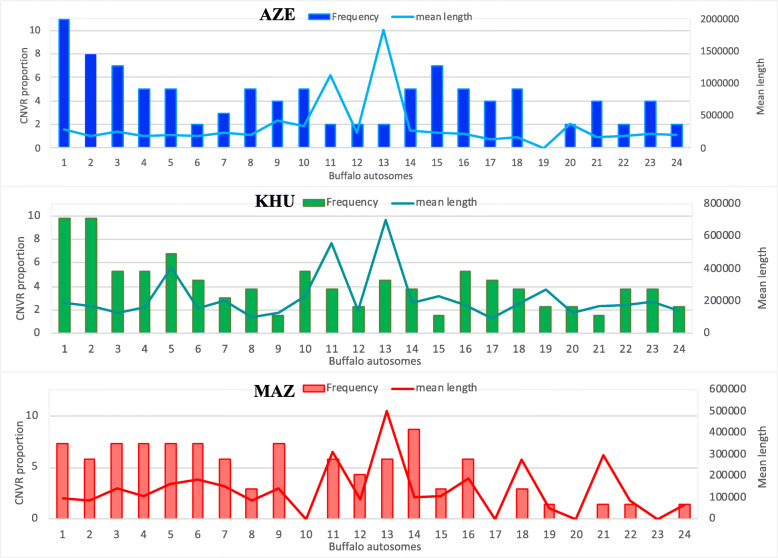
Frequencies (columns) and mean length in Mb (line) of CNVRs (identified in more than 5% of samples) for each chromosome for each of the three breeds: Blue – AZE; Green – KHU; Red – MAZ

For each population, a graphical representation of CNVRs frequencies on autosome is shown, together with the mean CNVRs coverage length (Fig. 2). The number of detected CNVRs among chromosomes is uneven, and no correlation between chromosomes length and mean CNVRs length resulted in these populations. The mean CNVRs length is not uniform along all chromosomes, mainly for MAZ breed. Two peaks (mean length) are evident for all breeds: on chromosomes 13 the mean CNVRs lengths were about five (AZE), and three (KHU and MAZ) times higher respect to the total mean length calculated for all autosomes. The second peak, found on chromosome 11, is lower respect to the previous one, but observable in all breeds.

**Fig. 3:**
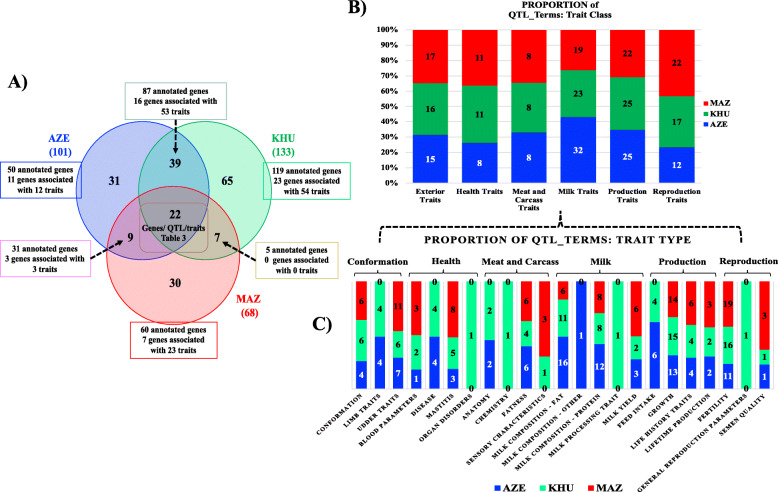
**a** Venn diagram of pop_CNVRs and common_CNVRs and count of genes associated with QTL_Terms (Trait Type and Trait Class) for each buffalo breed (Blue – AZE; Green – KHU; Red – MAZ); **b** Proportion of QTL_Terms (trait class) and **c** proportion of QTL_term (trait type) for each of the three breeds (Blue – AZE; Green – KHU; Red – MAZ) referred to association studies available for bovine species (https://www.animalgenome.org/cgi-bin/QTLdb/BT/index, accessed on 30 October 2020)

Within the 203 non redundant CNVRs a different proportion of common_CNVRs were observed among the three populations (Fig. 3): i) MAZ shared the 45.6% of AZE regions (corresponding to 30.7% of AZE regions); ii) MAZ shared the 42.6% of KHU regions (corresponding to the 23.3% of KHU regions); iii) AZE shared the 60.4% of KHU regions (corresponding to the 45.8% of KHU regions). Among the common_CNVRs, 22 are those identified in all three populations (Table 3).

**Table 3:** Details of the 22 common_CNVRs identified in all three Buffalo breeds. Number of samples together with annotated genes and QTL_Terms (Trait Names) and IDs – traits are also reported. QTL are in concordance with Animal Genome Cattle QTL Database

Chr	Start	End	State	TOT	AZE	KHU	MAZ	Gene	QTL_Terms: Trait Names
chr1	26,703,602	26,982,884	gain	**84**	65	15	4		
chr1	152,804,323	152,948,116	loss	**28**	21	5	2	LOC102407618	
chr2	3,733,628	4,072,437	complex	**29**	19	7	3	LY86	
chr2	101,529,318	101,684,916	complex	**23**	13	8	2		
chr3	31,955,474	32,522,879	complex	**84**	53	29	2	ELAC2, ARHGAP44, MYOCD	
chr3	114,751,926	114,833,959	loss	**40**	23	15	2	PCSK5	
chr4	153,409,610	153,553,690	complex	**38**	27	8	3		
chr5	56,890,564	57,111,573	loss	**63**	36	25	2	HLX, MARC1, MARC2	
chr5	33,931,170	34,500,134	complex	**35**	24	9	2	RPL22, RNF207, ICMT, HES3, GPR153, ACOT7, HES2, ESPN, TNFRSF25, PLEKHG5, NOL9, TAS1R1, ZBTB48, KLHL21, PHF13, THAP3, DNAJC11, CAMTA1	ACOT7: Somatic cell score – QTL:157327; TAS1R1: Height (24 months) – QTL:20633, 20,630; Hip height – QTL:20629, 20,631.
chr6	10,583,089	11,004,788	loss	**30**	16	8	6	LOC102415566, LOC102415903, LOC102416230, LOC102416552, LOC102389114, LOC102389455, LOC102413384, SPTA1, LOC102414242, LOC112585453, LOC102391442, LOC102414573, LOC102391758	
chr7	78,061,564	78,226,918	loss	**55**	35	18	2		
chr7	102,880,560	103,212,455	complex	**23**	14	7	2		
chr8	38,899,124	39,035,018	gain	**157**	91	52	14		
chr11	78,128,432	80,062,848	complex	**59**	31	21	7	LOC102389658 (ADSL-like), LOC112587962	
chr13	14,124,616	17,645,807	complex	**209**	150	50	9	CLDN10, ABCC4-like, OCIA-like	
chr13	26,851,401	27,052,499	complex	**39**	29	8	2		
chr14	52,975,663	53,315,557	complex	**75**	43	26	6		
chr14	67,343,425	67,561,999	complex	**55**	38	15	2	LOC102389323, ECHDC3, USP6NL	
chr16	49,791,674	50,216,803	complex	**131**	101	23	7	ABCC8, USH1C, MYOD1, OTOG, KCNC1, SERGEF	
chr18	60,924,568	61,091,446	loss	**69**	44	18	7	cationic amino acid transporter 3-like	
chr21	54,520,603	54,985,963	complex	**36**	15	19	2	SLC6A11, ATP2B2	
chr24	37,455,258	37,684,596	complex	**23**	15	6	2	RBFOX1	RBFOX1: Dopamine level – QTL:194894, 194,895, 194,896; Sperm counts – QTL:62089.

The pop_CNVRs represented about half of total CNVRs number, except for AZE for which the common_CNVRs are more than twice that the pop_CNVRs.

A total of 234, 365, and 158 genes were annotated within AZE, KHU and MAZ CNVRs, respectively (corresponding to 409 non redundant buffalo genes) (Supplementary Table S3) and their functional classification according to DAVID database is reported in Supplementary Table S4 and in Table 4 (Nominal *P*-value < 0.10).

**Table 4:** Gene annotation according to DAVID Database (Functional Annotation Clustering tool) (*P*-Value < 0.10). Species used as genetic background: *Bos taurus;* list uploaded = n.409 genes; genes recognized = n.334). Bold = *P*-value < 0.05

Category	Term	N	***P***-value	Genes
Biological Process	GO:0000381 ~ regulation of alternative mRNA splicing, via spliceosome	5	**3.47E-03**	RBFOX1, MYOD1, MAGOH, RBM11, SRSF12
	GO:0042060 ~ wound healing	5	**3.85E-03**	SLC11A1, TFF3, TFF2, MIA3, TFF1
	GO:0006811 ~ ion transport	4	**6.04E-03**	CLDN10, SLC11A1, SLCO2B1, CLDN16
	GO:0032691 ~ negative regulation of interleukin-1 beta production	3	**6.39E-03**	GHSR, ACP5, TNFAIP3
	GO:0030163 ~ protein catabolic process	5	**7.31E-03**	PAG8, PAG18, PAG17, PAG19, PAG16
	GO:0050885 ~ neuromuscular process controlling balance	5	**8.58E-03**	RBFOX1, JPH3, CAMTA1, ATP2B2, NBN
	GO:0021952 ~ central nervous system projection neuron axonogenesis	3	**1.07E-02**	SPTBN4, EPHB1, EPHB3
	GO:0007605 ~ sensory perception of sound	6	**2.42E-02**	SPTBN4, CNTN5, TMPRSS3, SLC52A3, OTOG, USH1C
	GO:0070328 ~ triglyceride homeostasis	3	**3.28E-02**	SCARB1, HNF4A, ANGPTL8
	GO:0006508 ~ proteolysis	8	**3.93E-02**	CAPN14, DPP6, CPXM2, PAG8, PAG18, PAG17, PAG19, PAG16
	GO:0018108 ~ peptidyl-tyrosine phosphorylation	3	**4.52E-02**	RIPK2, MAP 2 K6, IGF1R
	GO:0021915 ~ neural tube development	3	5.42E-02	NUP133, INTU, PLXNA2
	GO:0051480 ~ regulation of cytosolic calcium ion concentration	3	5.89E-02	CALB1, ATP2B2, TMEM64
	GO:0032465 ~ regulation of cytokinesis	3	5.89E-02	UVRAG, KLHL21, BIRC6
	GO:0006915 ~ apoptotic process	8	7.69E-02	KANK2, PEG3, C1H3ORF38, RIPK2, JADE1, ARRB1, BIRC6, MAP 2 K6
	GO:0007586 ~ digestion	3	7.92E-02	TFF3, TFF2, TFF1
	GO:0010508 ~ positive regulation of autophagy	3	7.92E-02	UVRAG, TRIM65, TRIM21
Cellular Compounent	GO:0005737 ~ cytoplasm	64	6.17E-02	DOCK6, JADE1, TNFAIP3, ARRB1, ECSIT, IGF1R, ARHGAP44, MAP 1LC3B, CATIP, HNF4A, MAGOH, GDAP1L1, PRX, TRIM21, RBFOX1, PEG3, RIPK2, CLDN10, ACTA1, BIN1, EIF4E1B, RUFY3, RNF207, SHPK, TFF3, TAX1BP3, DNAJC9, BIRC6, ARHGEF5, SNRPA, KANK2, NUMBL, WDR26, INTU, ZBTB48, USH1C, NEDD4L, GLRX, EVPL, STK3, UBE2J1, PDLIM1, NEU3, PDZD2, FGGY, SERTAD1, HECTD2, TRIM47, SPOCK1, CAMTA1, MAP 4 K5, MAP 2 K6, HIPK4, ECD, LIMK1, KLHL21, HTR1B, ELL2, WAPL, CAPN14, SPSB1, PPP1R1C, SERGEF, GALK1
	GO:0043005 ~ neuron projection	6	6.94E-02	SLC4A8, GHSR, CALB1, LIMK1, SLC17A6, SLC6A11
	GO:0000139 ~ Golgi membrane	8	7.51E-02	GALNT6, SEC16B, EXT2, GOLPH3, GALNT14, MAN1A1, MIA3, GALNTL6
	GO:0005578 ~ proteinaceous extracellular matrix	7	8.27E-02	OLFML2B, ENAM, COL22A1, TFF3, LTBP4, COL6A3, SPOCK1
Molecular Function	GO:0004190 ~ aspartic-type endopeptidase activity	*5*	**5.14E-03**	PAG8, PAG18, PAG17, PAG19, PAG16
	GO:0005524 ~ ATP binding	*30*	**4.47E-02**	ATP6V1A, NVL, RHOBTB3, NOL9, IGF1R, STK3, HK3, AKT2, FYN, MAP 4 K5, EPHB1, GLUL, EPHB3, MAP 2 K6, HIPK4, ABCC4, RIPK2, RFC2, ABCC8, INSR, LIMK1, ATP2B2, CIT, ACTA1, BBS10, CDK3, ROR1, ABCG1, GALK1, CDKL1
	GO:0016874 ~ ligase activity	5	7.75E-02	MCCC2, HECTD2, NEDD4L, TRIM21, GLUL
	GO:0001105 ~ RNA polymerase II transcription coactivator activity	3	8.75E-02	MYOCD, WBP2, JADE1
KEGG	bta04360:Axon guidance	8	**2.61E-03**	EPHA6, UNC5A, DCC, LIMK1, PLXNA2, FYN, EPHB1, EPHB3
	bta04024:cAMP signaling pathway	7	7.61E-02	GHSR, HTR1F, ACOX1, AKT2, ARAP3, HTR1B, ATP2B2
	bta04520:Adherens junction	4	8.14E-02	INSR, FYN, SORBS1, IGF1R
	bta00512:Mucin type O-Glycan biosynthesis	3	8.24E-02	GALNT6, GALNT14, GALNTL6

The Animal Genome Cattle Database was accessed to reveal the potential relationships between CNVRs and QTL. As shows in Fig. 3, in Table 3, and in Table 5, 59 genes resulted associated with a total of 80 different “Trait Name”, grouped in 23 “Trait Type” (Anatomy, Blood parameters, Chemistry, Conformation, Disease, Fatness, Feed intake, Fertility, General reproduction parameters, Growth, Life history traits, Lifetime production, Limb traits, Mastitis, Milk composition – fat, Milk composition – protein, Milk processing trait, Milk composition – other, Milk yield, Organ disorders, Semen quality, Sensory characteristics, Udder traits) corresponding to six “Trait Class” (Exterior, Healthy, Meat and Carcass, Milk, Production, and Reproduction Traits) according to Animal Genome Database Cattle Traits nomenclature.

**Table 5:** Common and pop_CNVRs for which genes and QTL annotation were available. Complete list of common and pop_CNVRs and QTL_IDs (referring to *Bos taurus* species) are reported in Supplementary Table 3. QTL_Terms: Trait Names are grouped in Fig. 3 as Traits Type according to Animal Genome Cattle QTL Database. The Table does not include common_CNVRs reported in Table 3

Breed	Chr	Start	End	State	N	Gene	QTL_Terms: Trait Names
**AZE**	1	186,836,088	187,487,608	complex	14	PDE9A	Bovine respiratory disease, Milk fat yield susceptibility, Milk protein yield, Milk yield
						SLC37A1	Milk phosphorus content
	1	20,942,758	21,126,649	loss	16	PRAG1	Bovine tuberculosis susceptibility
	2	57,805,999	58,006,839	gain	18	BIN1	Residual feed intake
	8	26,033,634	26,136,325	complex	13	SNX13	Milk protein percentage
	10	72,266,178	72,898,604	complex	16	FAM184A	Subcutaneous fat
	12	15,024,107	15,144,657	loss	13	TTC27	Milk fat yield
	15	78,109,455	78,335,826	loss	12	COL22A1	Milk fat percentage, Milk protein percentage, Milk fat yield, Milk yield, Milk protein yield
	15	8,242,728	8,744,577	loss	13	DECR1	Fat thickness at the 12th rib
	15	16,595,357	16,770,941	gain	14	STK3	Milk fat yield, Milk protein percentage, Milk protein yield
	16	21,588,533	21,771,264	gain	18	WT1	Carcass weight, Milk fat percentage
**KHU**	2	70,740,089	70,803,405	gain	5	TTN	Udder cleft
	2	159,382,105	159,476,051	loss	5	SLC11A1	Bovine tuberculosis susceptibility, M. paratuberculosis susceptibility
	3	38,990,851	39,088,431	gain	5	ITGAE	Milk conjugated linoleic acid content
	5	36,870,251	37,136,533	gain	7	SPSB1	Milk protein yield, Somatic cell score
	5	98,013,455	98,218,453	loss	6	ANO5	Milk C14 index, Curd firming rate, Meat texture
	6	26,325,308	26,500,901	gain	8	CD2	Calving to conception interval, Inseminations per conception, Milk fat percentage, Net merit, Somatic cell score
	7	31,368,913	31,752,823	loss	5	RUFY3	Milk protein percentage, Milk protein yield, Milk yield, Residual feed intake, Somatic cell score
	8	116,160,601	116,290,580	gain	6	DPP6	Average daily gain, Body weight (birth), Body weight (weaning), Bovine respiratory disease susceptibility
	9	62,716,777	62,830,823	loss	5	SPOCK1	Body depth, Calving ease (maternal), Calving ease, Conception rate, Daughter pregnancy rate, Feet and leg conformation, Foot angle, Length of productive life, Milk fat percentage, Milk fat yield, Milk protein percentage, Milk protein yield, Milk yield, Net merit, PTA type, Rear leg placement - rear view, Rump width, Somatic cell score, Stillbirth, Udder attachment, Udder depth
	10	90,708,913	91,158,966	gain	5	CD109	First service conception, Inseminations per conception
	15	51,487,540	51,850,347	complex	5	CRH	Average daily gain, Carcass weight, Conformation score, Connective tissue amount, Longissimus muscle area, Marbling score, Muscle pH, Subcutaneous fat
						TRIM55	Carcass weight
	16	10,532,220	10,757,353	loss	5	ACCS	Serotonin level
						ACCSL	Conception rate, Serotonin level
						ALKBH3	Stayability
	17	17,338,207	17,457,683	gain	6	CIT	First service conception QTL, Inseminations per conception
	17	21,924,791	22,056,117	complex	6	SCARB1	Bovine respiratory disease susceptibility, Milk beta-carotene content, Milk fat percentage, Milk protein percentage
	18	39,524,271	39,714,171	loss	6	HYDIN	Marbling score
	18	64,081,682	64,220,127	complex	5	PEG3	Body depth, Fat cover, Rump width, Stature, Stillbirth
							Angularity, Body depth, Stature, Stillbirth
	19	41,270,588	41,632,848	gain	6	PDZD2	Somatic cell score
	20	61,571,126	61,672,190	gain	8	IGF1R	Age at puberty, Body size, Body weight (birth), Body weight (weaning), Milk protein yield, Milk yield, Milk protein percentage, Milk fat yield, Milk fat percentage, Inseminations per conception, Bovine respiratory disease susceptibility, Body weight (yearling)
	22	4,920,880	5,171,378	gain	5	NEDD4L	Abomasum displacement
**MAZ**	3	133,069,378	133,117,242	gain	2	LOXL2	Milk fat yield
	6	96,104,850	96,210,725	loss	3	AGBL4	Milk protein yield, Milk yield
	6	81,057,321	81,285,031	loss	2	ROR1	Body depth, Calving ease (maternal), Calving ease, Daughter pregnancy rate, Length of productive life, Milk protein percentage, Net merit, PTA type, Rump width, Somatic cell score, Stature, Stillbirth (maternal), Stillbirth, Strength, Udder attachment, Udder depth, Udder height
	9	15,430,969	15,568,337	gain	2	ELL2	Milk fat yield, Milk protein yield, Milk yield, Somatic cell score
	12	44,167,548	44,199,735	loss	2	SH3RF3	Teat placement
	18	49,428,499	49,811,006	gain	2	NUMBL	Shear force
							Conception rate, Daughter pregnancy rate
**AZE KHU**	1	139,476,110	139,719,306	loss	26	FNDC3B	Calving ease (maternal), Daughter pregnancy rate, Length of productive life, Milk protein yield, Rear leg placement - side view, Somatic cell score, Teat length, Dairy form, Dry matter intake, Milk protein percentage, Net merit, Residual feed intake, Stillbirth, Udder cleft, Calving ease
						GHSR	Average daily gain, Body weight (slaughter), Body weight (test end), Carcass weight
						TNFS10	Interval from first to last insemination, Milk fat percentage, Milk fat yield, Milk protein yield
	3	7,562,702	7,799,466	gain	22	ACOX1	Marbling score, Subcutaneous fat, Subcutaneous fat
	3	67,310,421	67,462,093	complex	18	GALNTL6	Bovine tuberculosis susceptibility
	3	68,223,946	68,369,724	gain	23	GALNTL6	Bovine tuberculosis susceptibility
	4	91,862,787	92,034,556	gain	68	GALNT6	Milk linoleic acid content, Udder cleft, Udder texture
	11	12,884,241	13,195,697	complex	20	NRXN3	Milk protein yield
	14	10,786,055	10,971,651	complex	28	HNF4A	Body length, Body weight (24 months), Chest girth, Height (24 months)
	16	30,256,354	30,582,499	complex	46	ARRB1	Bovine respiratory disease susceptibility, Milk fat percentage, Milk fat yield, Milk yield
						NEU3	Conception rate, Daughter pregnancy rate, Net merit, Length of productive life
	16	75,669,845	75,781,021	complex	42	CNTN5	Body depth, Calving ease (maternal), Calving ease, Feet and leg conformation, Foot angle, Milk fat percentage, Milk fat yield, Milk protein percentage, Milk protein yield, Net merit, PTA type, Rear leg placement - rear view, Residual feed intake, Rump width, Somatic cell score, Stature, Stillbirth, Strength, Udder attachment, Udder depth, Udder height
	18	12,571,250	12,842,177	complex	37	ZCCHC14	Inseminations per conception
	20	40,344,161	40,510,307	gain	69	PCSK6	Metabolic body weight, Residual feed intake, Dry matter intake
	23	16,558,338	16,817,683	complex	26	PDLIM1	Rump angle
	23	18,714,144	18,844,893	complex	30	CRTAC1	Milk C14 index, Milk fat yield, Milk myristoleic acid content
**AZE MAZ**	8	70,758,890	71,075,937	complex	14	OSBPL3	Fat thickness at the 12th rib
	9	94,954,953	95,685,910	loss	17	ANGPTL8	Body length
						ARHGEF18	Fat area to ribeye area ratio

**Fig. 4:**
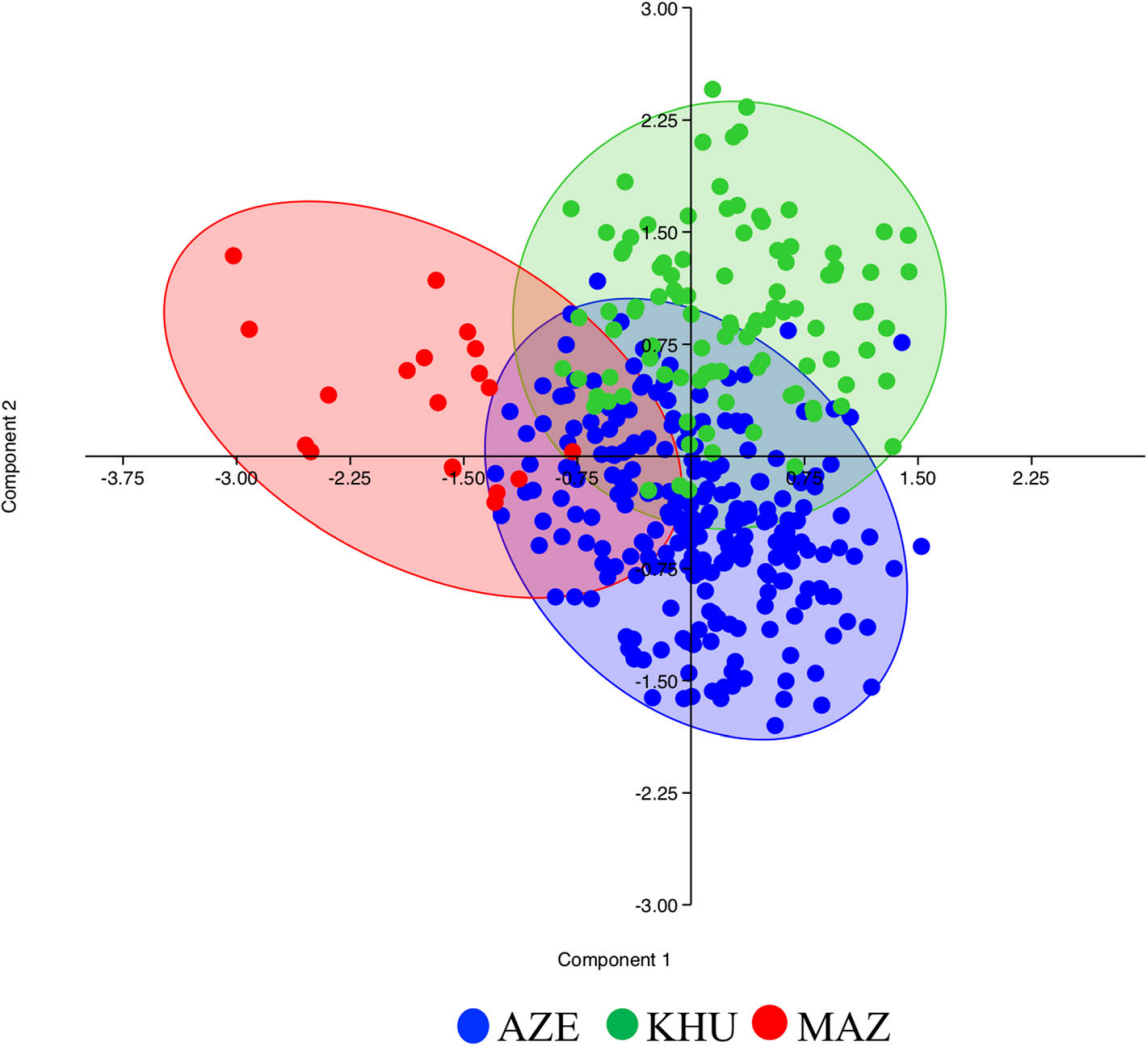
CNVRs Principal Component Analysis (PCA) graph: Blue – AZE; Green – KHU; Red – MAZ

Results of PCA showed a spatial distribution of samples (Fig. 4) due to the difference in CNV across the three pop_CNVRs. This is reflecting the overlapping among common_CNVRs and proprietary CNVRs or each breed as shown in Fig. 3 (A) in the Venn diagram.

## Discussions

In Iran, breeding of river buffaloes plays an important role in the economy of the country, also impacting on social and cultural activities [7]. In this country the most common local buffalo breeds are the Azeri and Khuzestani. The Mazandrani breed is also farmed in Iran, but its numeric consistency of about 4000 individuals is lower respect to those reported for AZE and KHU breeds (i.e. 119,000 and 81,000 buffaloes, respectively) [4]. All these three breeds, different in morphology, have been undergone a different selection processes and are well adapted to different environments proper of the geographical areas of the country in which they are reared: Azari – the north-west-north of Iran (70%), Khuzestani – west and south-west (22%) of the Country while the Mazandarani is farmed in the north (8%) region (http://www.fao.org/3/ah847e/ah847e00.pdf).

The genetic variability in Iranian buffalo breeds have been recently studied and findings based on the Linkage Disequilibrium (LD) obtained from the Affymetrix 90 K SNP genotypes [19] showed a close genetic relation between AZE and KHU due to a high LD consistency across the two populations and a lower similarity when the comparison involved MAZ with both AZE and KHU due to variability in the LD trend within breed. These results are consistent with those found in an additional Principal Component Analysis (Supplementary Figure S1) based on SNPs genotypes showing a clear clustering of the three breeds. Additionally, the F_ST_ statistics here calculated as in [3], shows a differentiation among the three breeds with similar F_ST_ values: the lowest between AZE vs KHU (F_ST_ = 0.017 our study, F_ST_ = 0.021 [3]); an intermediate between AZE vs MAZ (F_ST_ = 0.045 our study, F_ST_ = 0.038 [3]); the highest between KHU and MAZ (F_ST_ = 0.058 our study, F_ST_ = 0.045 [3]).

To improve the knowledge on these populations, we investigated the genomic structure of the Iranian buffaloes through the analysis of the CNVs, in order to provide additional information that could be used for breeding and conservation programs of these populations, as concluded by Clop et al. [20].

A genome wide CNVs detection has been here performed resulting in a total of 9550 CNVs and 2.995 CNVRs in the three breeds. The AZE and KHU show about 50% of their CNVR as singleton, while MAZ breed 73%. The large singleton_CNVRs proportion for MAZ is most likely due to the reduced sample size that does not permit to identify a larger proportion of pop_CNVRs in this breed. Excluding singleton_CNVRs the CNVRs resulted 1605 in the three breeds. Among the 1605 pop_CNVRs, 302 were identified across breeds in at least 5% of individuals within breed, covering about 1.97% of the buffalo genome (calculated considering the no redundant 203 CNVRs). Out of these latter CNVRs, 22 (10.8%) are those shared by all populations, identifying a common genomic structural background of among these buffaloes. In fact, when considering the common_CNVRs counts (those shared among breeds), the highest number of common_CNVRs are found in AZE having the lowest proportion of pop_CNVRs (30.7%) respect to those identified for KHU (48.9%) and for MAZ (44.1%). MAZ breed shared a higher number of CNVRs with AZE respect to those resulted in common with KHU. This result suggest that the moderate level of admixture identified for MAZ and AZE by [6], is confirmed here and graphically visualizable in the PCA (Fig. 4), where MAZ buffaloes are mainly distributed close to AZE individuals. The PCA showed in Fig. 4 is also showing that CNVs are not the same across the three breeds as they do partially overlap: in fact only 22 CNVRs are in common among the three breeds, while 31, 65 and 30 were found only in the AZE, KHU and MAZ respectively, as showed by Venn diagram in Fig. 3. CNVs are in fact a different class of markers respect to SNP and it is expected that PCA is discriminating the population not in the same exact manner.

The most represented common_CNVR, defined by the CNVs identified in 209 buffaloes of all the three breeds (Table 3), mapped on chr13. It does not show a specific CNV pattern of state in a particular breed, i.e. only gain or only loss, but is having a complex behavior. A part of this region harbors 21 LOC gene IDs annotated consecutively over the genome (i.e. from 14,252,993 to 17,645,518 bp), all correspond to the same multidrug resistance-associated protein 4-like protein coding gene, also known as ATP-binding cassette sub-family C member 4 (*ABCC4*). This gene appears to be involved in several basic metabolic pathways including resistance/susceptibility to intestinal nematodes [21], feed efficiency [22], and marbling score [23]. Recently, in a study conducted on dairy buffaloes, for the *ABCC4* gene it has been also highlighted its possible contribution on reproduction traits, resulting among the top genes associated with number of services per conception [24]. Additionally, the expression of *ABCC4* gene increases in pregnant cattle and pigs’ endometrium and it could be important to support pregnancy given its role in prostaglandin efflux from cells [25, 26].

Other two common_CNVRs were defined by CNVs called in more than 100 individuals. The first region mapped on chr8 (at 38,899,124-39,035,018 pb) and the second one on chr16 (at 49,791,674-50,216,803 bp). Only this latter region, resulted complex in AZE and KHU and gain in MAZ, harbors genes (*ABCC8, USH1C, MYOD1, OTOG, KCNC1, SERGEF*), which all resulted close (i.e. surrounding 200 kb) to the significant SNPs affecting Nellore age at first calving [27]. Also, the *MYOD1* gene is a component of myogenic regulatory factors involved in myoblast differentiation and in concordance with [28, 29] results, this gene may potentially affecting meat production.

The CNVRs in which genes or QTL were not annotated are those identified in lower number of individuals (within breed). For these samples the CNVs defining CNVRs also resulted with a large proportion of complex state (21% for AZE; 17% for KHU, and 3% for MAZ, excluding singleton_CNVRs.

Recent literature support that CNV may have a role in selection mechanism in addition to SNPs and that CNV changes may indicate that artificial selection may cause difference in genetics and phenotypes among breeds [30, 31]. We could speculate that the lack of a strong directional selection for a specific trait did not favor the increase in copies for specific genes as occurred in human [32], dogs [33] or polar bears [34], where the dietary shift produced an increase in DNA copy number where the *AMY* gene family was involved, an example of positive selection on CNV. In livestock populations where the directional selection is not focused towards a unique direction or for a specific trait, the proportion of complex CNVRs is comparable to the ones here found. This is the case of creole cattle in Mexico where the proportion of complex CNVRs was 16% [35] and of the Aosta Red Pied (Valdostana Red Pied) where the selection goal contemporarily focuses on milk, meat and adaptation to summer pasture practice, i.e. adaptation to harsh environment [36]. A further evidence is provided in the avian species as for strongly selected populations, as in chicken, where a very low proportion of complex CNVRs, 0 to 5% was found, while in a the non-selected Mexican creole poultry population the proportion of complex CNVRs was up to 14% [14].

Within the CNVRs here found in gain in state (i.e. defined by CNVs all duplicated), several annotated genes with a well-known associated phenotype in cattle or in other species were found in addition to those reported in Tables 3 and 5. Regarding the *PCSK6* gene there are evidences of an association with follicle development in human [37], while for *GALNTL6* with feed efficiency and growth traits in cattle [38], with saturated fatty acids profile in intramuscular fat of the longissimus thoracis muscle of Nellore [39], and with cow and heifer conception rate [40]. For the same breeds, gain regions also harbored *GALNT6* and *FYN* genes, both involved in reproduction traits in cattle (https://www.teagasc.ie/media/website/publications/2010/FertilityGeneExpression_5517.pdf) and in mouse [41], respectively. The *FYN* gene, considered by [42] as thermotolerant gene, seems to have a role in cow conception and early embryo development in cattle. Finally, *PCSK6* and *SNRPA1,* together with *PLXNA2,* mapping within an AZE gain CNVR, resulted lying within a positive selection signature region identified using SNP as genetic markers in creole breeds [30]. The *PLXNA2* gene has been also associated with cattle temperament [43]. For the MAZ breed, there are few gain CNVRs harboring genes: this can be related to the very low number of samples available in this study. Among these regions, the one located on chr1 overlaps *EPHB3*, a gene resulted associated with muscling at weaning (MW) and muscling at yearling (MY) in *Bos indicus* populations [44]. Also, *EPHB3* maps in a selection signature region identified by V_ST_ analysis based on CNVs performed through a comparison between Valdostana Red Pied vs Italian Brown Swiss, a double proposal and a dairy cattle breed, respectively [36].

According to the annotation analysis performed with DAVID Database, buffalo CNVRs are enriched in genes (*n* = 334 recognized IDs) mainly involved general biological processes (Supplementary Table S3). Also, a total of 80 different “QTL_Trait-Terms” associated with 59 genes have been identified and classified in 6 major QTL trait categories (Fig. 4 and Tables 3 and 4), of which the most represented are Milk (i.e. Milk composition – fat and –protein) and Production Traits (i.e. Growth) (Tables 3 and 4, Supplementary Table S3). We did not observe a prevalence of a particular QTL_Trait Term (taking in to account the differences in CNVRs and annotated genes counts) in one of the three breeds, except for AZE – General Milk– and for MAZ – Reproductive QTL_Trait Terms.

## Conclusions

The knowledge of genomic variation in the water buffalo species is still very limited and most of the recent findings still rely on comparison with cattle species. This work provides a step forward in the biological interpretation of genomic variation in the buffalo species. As the CNVs are known to be mostly non-neutral markers, these results may contribute to interpret genomic variation within and among the buffalo populations, that can be used to provide insights about their recent selection and adaptation to environment.

We may speculate that the presence of the set of genes and QTL traits harbored in the CNVRs here mapped could be linked with the buffalo’s natural adaptive history, i.e. to their ability to adapt to diverse and severe environmental conditions (different for AZE and MAZ respect to KHU) and may be occurred because these bovid in recent time have started a selection program for milk yield, that is a primary food source from this species.

## Methods

### Sampling

A total of 384 Iranian Buffalo raw genotyping data (i.e. cel.files) obtained using the Axiom® Buffalo Genotyping Array 90 K (Affymetrix, Santa Clara, CA, USA) were available for the three breeds [4, 6, 9] and utilized as input files in order to obtain the Log R Ratio (LRR) values for each sample. As extensively described in [4] and in [19], the SNPs data comes from populations sampled with the aim to be representative of each breed. The AZE breed (252 samples) was sampled in the East and West-Azarbaijan, Ardebil and Gilan provinces, the KHU (110 samples) in Khuzestan and Kermanshah provinces, and the MAZ (22 samples) from Miankaleh wildlife sanctuary of Mazandaran province.

A quality control of raw intensity files using the standard protocol in the Affymetrix Power Tools package (www.affymetrix.com) was performed in order to guarantee a high quality of obtained data. Individuals with a value of call rate less than 97% and Dish Quality Control less than 82% were removed. After quality control a total of 9 low quality samples have been identified and not used.

The marker positions of the Genotyping Array 90 K array were recently updated and based on the newly released University of Adelaide water buffalo assembly (UOA WB v. 1; https://www.ncbi.nlm.nih.gov/assembly/GCF_003121395.1).

A total of 35,114 SNPs was filtered out, in part for their undefined chromosomal locations, and in part because a proportion of SNPs had more than one nearby probeset: for these latter the Axiom Analysis suite picked the best performing probeset per each SNP. A total of 70,230 SNPs was then retained in the analysis mapped on the *Bubalus bubalis* (UOA_WB_1) genome assembly.

To confirm the clustering of individuals to the three populations, the genetic diversity within and among breeds was explored using SNP genotypes by Principal Component Analysis (PCA) and by the pairwise Fixation Index obtained according to the pipelines of Golden Helix (SVS) 8.8.4 software (Golden Helix Inc., Bozeman, MT, USA).

This study did not require approval from the Animal Care and Use Committee as we use already available data obtained in previous researches compliant [4, 6, 9] with rules and regulations for animal sampling.

### CNVs, CNVRs detection and subsequent analyses

The CNV detection was performed on the 24 autosomes, using the Copy Number Analysis Module (CNAM) of SVS (Golden Helix, Bozeman, MT, USA), through the univariate analysis based on LRR values obtained using the Axiom® CNV Summary Tool software (www.affimetrix.com). A quality assurance of LRR raw data and filtering of outlier samples was performed before CNV calling with the SVS software through: i) the overall distribution of derivative log ratio spread (DLRS) values as described by [45]; ii) the GC-wave factor (GCWF) that measures the GC-content causing the fluctuation of a signal intensity file [46]. A total of 14 samples were excluded during the quality assurance because of their high DLRS and GCWF values. Consequently, the CNV mapping was performed on a final dataset of 361 samples (n. 242 – AZE, n. 100 – KHU, and n. 19 – MAZ) including as parameters in CNAM: maximum 100 segments per 10,000 markers; minimum of 3 marker per segment; 2000 permutations per pair with a *p*-value cut off of 0.005.

CNV regions (CNVRs) at population level were obtained by merging CNVs that overlapped by at least 1 bp in at least two animals using the -mergeBed command of Bedtools [47]. CNVRs were then cataloged as gain, loss and complex (i.e. CNVRs comprising both gain and loss CNVs) regions. A CNV identified in only one individual was classified as singleton_CNVR. Finally, the “-intersectBed” command of Bedtools software was employed to catalogue CNVRs as “pop_CNVRs” and “common_CNVRs” if they have been mapped in only one population (no intersection among CNVRs identified in more than one breed) or they resulted in common among breeds (part – at least 50% – or full overlapping), respectively. Only CNVRs found in at least 5% of the individuals of a breed were considered to infer statistics at population level and for the gene annotation.

The complete list of buffalo protein coding genes was downloaded from NCBI online Database (https://www.ncbi.nlm.nih.gov/genome/browse/#!/proteins/791/374666|Bubalus%20bubalis/). Genes with official “gene name ID” and LOC genes associated with a protein coding gene name (excluding uncharacterized ones) were annotated within the detected CNVRs using the Bedtools “-intersectBed” command. Gene Ontology terms (GO) and Kyoto Encyclopedia of Genes and Genomes (KEGG) pathway analyses were performed using the DAVID Bioinformatic Database (https://david.ncifcrf.gov).

As the Quantitative Trait Loci (QTL) database for the buffalo species is not available, the QTL associated to the genes here found in the CNVRs were identified in the cattle QTL database (QTLdb: https://www.animalgenome.org/cgi-bin/QTLdb/BT/search) by gene name, using the “Search by associated gene” option of QTLdb.

In order to disclose diversification of the three buffalo breeds based on CNVs, a Principal Component Analysis (PCA) using Past software [48]. Sample-CNV genotypes were coded as “deletion”, “duplication”, and “normal” states for each of the identified CNVRs and used in the PCA analysis.

## Supplementary Information

**Additional file 1: Supplementary Table S1** List of CNVs identified for each Iranian river buffalo breed.

**Additional file 2: Supplementary Table S2** List of all CNVRs identified for each Iranian river buffalo breed.

**Additional file 3: Supplementary Table S3** List of 203 non redundant CNVRs. Samples count for each breed, genes and QTL are also reported.

**Additional file 4: Supplementary Table S4** Gene annotation according to DAVID Database.

**Additional file 5: Supplementary Figure S1** PCA and F_ST_ based on SNP genotypes.

## Data Availability

The datasets supporting the results and conclusions of our study are included within the article and in the additional files.

## Abbreviations

SNPs: Single Nucleotide Polymorphisms
QTL: Quantitative Trait Loci
CNVs: Copy Number Variants
CNVRs: Copy Number Variant regions
AZE: Azeri breed
KHU: Khuzestani breed
MAZ: Mazandrani breed
PCA: Principal Component Analysis
LD: Linkage Disequilibrium
