# Supplementary figures and images for: A genome-wide scan of copy number variants in three Iranian indigenous river buffaloes
Source: BMC Genomics. 2021 Apr 26;22:305. doi: 10.1186/s12864-021-07604-3 (PMC8077898; doi:10.1186/s12864-021-07604-3)

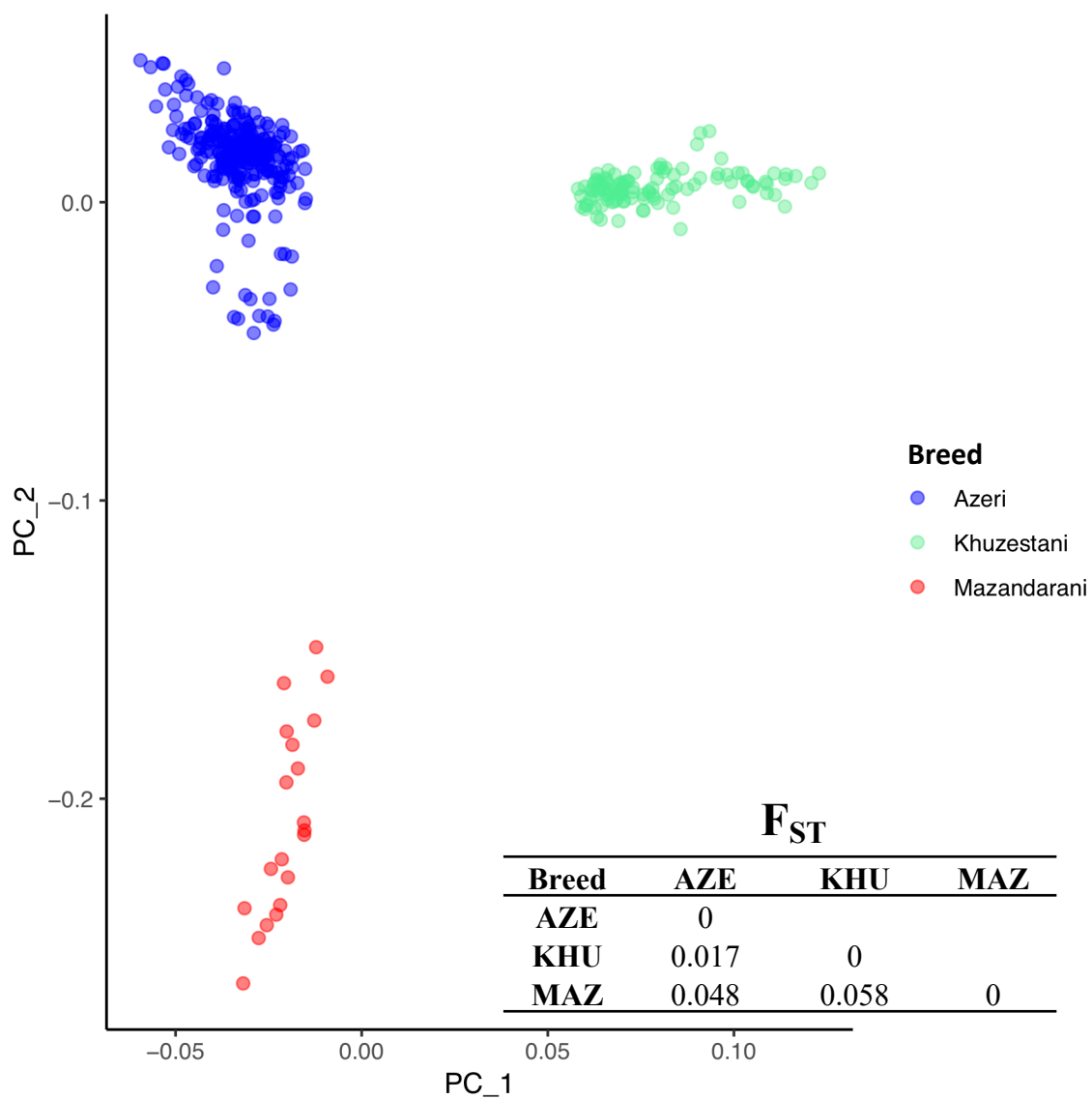

Supplement: Supplementary file 5 — Additional file 5: Supplementary Figure S1 PCA and FST based on SNP genotypes. [file 12864_2021_7604_MOESM5_ESM.pdf]
